# Effects of psilocybin versus escitalopram on rumination and thought suppression in depression

**DOI:** 10.1192/bjo.2022.565

**Published:** 2022-09-06

**Authors:** Tommaso Barba, Sarah Buehler, Hannes Kettner, Caterina Radu, Bruna Giribaldi Cunha, David J. Nutt, David Erritzoe, Leor Roseman, Robin Carhart-Harris

**Affiliations:** Centre for Psychedelic Research, Department of Medicine, Imperial College London, UK; Psychedelics Division, Neuroscape, Department of Neurology, University of California, San Francisco, USA

**Keywords:** Depressive disorders, pharmaceutical drug trial, randomised controlled trial, novel central nervous system drugs, antidepressants

## Abstract

**Background:**

Major depressive disorder is often associated with maladaptive coping strategies, including rumination and thought suppression.

**Aims:**

To assess the comparative effect of the selective serotonin reuptake inhibitor escitalopram, and the serotonergic psychedelic psilocybin (COMP360), on rumination and thought suppression in major depressive disorder.

**Method:**

Based on data derived from a randomised clinical trial (*N* = 59), we performed exploratory analyses on the impact of escitalopram versus psilocybin (i.e. condition) on rumination and thought suppression from 1 week before to 6 weeks after treatment inception (i.e. time), using mixed analysis of variance. Condition responder versus non-responder subgroup analyses were also done, using the standard definition of ≥50% symptom reduction.

**Results:**

A time×condition interaction was found for rumination (F(1, 56) = 4.58, *P* = 0.037) and thought suppression (F(1,57) = 5.88, *P* = 0.019), with *post hoc* tests revealing significant decreases exclusively in the psilocybin condition. When analysing via response, a significant time×condition×response interaction for thought suppression (F(1,54) = 8.42, *P* = 0.005) and a significant time×response interaction for rumination (F(1,54) = 23.50, *P* < 0.001) were evident. Follow-up tests revealed that decreased thought suppression was exclusive to psilocybin responders, whereas rumination decreased in both responder groups. In the psilocybin arm, decreases in rumination and thought suppression correlated with ego dissolution and session-linked psychological insight.

**Conclusions:**

These data provide further evidence on the therapeutic mechanisms of psilocybin and escitalopram in the treatment of depression.

Major depressive disorder (MDD) is one of the most burdensome disorders worldwide.^1^ Although its symptomatology is complex and heterogeneous, patients with MDD often engage in maladaptive coping strategies such as negative thought suppression and rumination, which interfere with effective problem-solving^2^ and emotional processing.^3^ Thought suppression is considered a defence mechanism characterised by the deliberate and effortful attempt to avoid distressing thoughts or memories.^3^ Although this may provide temporary relief, it generally precludes effective emotional processing and can lead to a higher recurrence of negative thoughts, paradoxically fuelled by suppression attempts.^4^

In the context of depression, rumination is defined as a rigid form of introspection characterised by ego-centric negative thoughts on one's ‘self’ and situation, as well as on the consequences and causes of such thoughts.^2^ Patients with depression often display deficient cognitive control resources and a negative cognitive bias that reflects and compounds their suppressive and ruminative tendencies. Thus, both thought suppression and rumination have been linked to the likelihood of maintaining, risk of recurrence and severity of MDD.^2,5^ Although thought suppression and rumination might appear clinically distinct coping strategies, previous research suggests that unsuccessful attempts to suppress negative thoughts may relate to an increased frequency of intrusive thoughts.^6^ This higher frequency of negative thoughts often coincides with ruminative loops, whereby intrusive thoughts consume attentional resources.

Currently the most common clinical treatment for MDD is antidepressant drugs, with selective serotonin reuptake inhibitors (SSRIs) like escitalopram being the most prevalent type. However, SSRI response rates are only around 50–60%, and side-effects such as sexual dysfunction and emotional ‘blunting’ are not infrequent.^7^ We recently conducted a randomised controlled trial (RCT) of 59 patients with MDD, comparing two doses of psilocybin – the most widely researched serotonergic psychedelic – administered 3 weeks apart, with 6 weeks of treatment with escitalopram (10–20 mg/d). Psilocybin was shown to be as effective as escitalopram in reducing depressive symptoms, but performed significantly better on measures of well-being, anhedonia, emotional acceptance, suicidality, and work and social functioning. However, the presence of motivated participants together with correct condition guessing might have biased findings in favour of psilocybin.^8^ The incidence of adverse events was similar in the trial groups, and no serious adverse events occurred. Psilocybin's side-effect profile was less diverse than that of escitalopram's, and superior in certain domains, including anxiety, dry mouth, sexual dysfunctional and emotional function.

Combined with psychological support, classic serotonergic psychedelics have been proposed to prompt a relaxation and potential revision of maladaptive cognitive and behavioural habits or biases, including negative beliefs about oneself and the world that are characteristic of MDD.^9,10^ This action has been linked to an enhancement of the complexity or entropy of spontaneous brain activity,^10^ neuroplasticity and psychological flexibility.^11^ Psychedelics have been shown to reduce negative appraisals,^12^ and qualitative reports from clinical research suggest a decrease in self-rumination and increase in acceptance of emotions after a psychedelic experience.^13^ However, no study to date has assessed the impact of psilocybin on clinical measures of rumination and thought suppression in an RCT with blinding procedures and an established antidepressant treatment as an active comparator. Here, we bridge this gap by using the 22-item Ruminative Response Scale (RRS)^14^ and 14-item White Bear Suppression Inventory (WBSI)^4^ in a trial of 59 patients with MDD treated with either psilocybin therapy or escitalopram.^8^

## Aims

This study sought assess the comparative effect of psilocybin and escitalopram on rumination and thought suppression. For reasons of parsimony and focus on the abovementioned psilocybin vs escitalopram trial, and because we felt these two outcome measures were sufficiently interesting and independent to warrant their own report, they were not analysed in the initial study report.^8^ Our primary hypothesis was that, compared with baseline, patients treated with psilocybin therapy will show a greater reduction in RRS and WBSI scores at the primary end-point relative to patients treated with escitalopram. Secondary hypotheses were that (a) psilocybin responders, defined in accordance with conventional criteria (i.e. a reduction of ≥50% in baseline symptom severity scores), would show significantly greater decreases in RRS and WBSI scores than escitalopram responders or non-responders in either condition; and (b) subjective effects linked to the psilocybin dosing sessions would correlate with changes in RRS and WBSI scores. A final analysis explored whether pre-trial discontinuation of SSRI medication affected results. These ancillary results can be found in the Supplementary Material available at https://doi.org/10.1192/bjo.2022.565.

## Method

The authors assert that all procedures contributing to this work comply with the ethical standards of the relevant national and institutional committees on human experimentation and with the Helsinki Declaration of 1975, as revised in 2008. All procedures involving human patients were approved by the Brent Research Ethics Committee, UK Medicines and Healthcare products Regulatory Agency (MHRA), Health Research Authority (HRA), Imperial College London Joint Research Office (JRO), General Data Protection Regulation (GDPR) (study reference: 17/LO/0389) and the risk assessment and trial management review board at the site (National Institute for Health and Care Research (NIHR) Imperial Clinical Research Facility). COMPASS Pathways provided psilocybin (as COMP360) upon receiving a Schedule 1 drug license from the UK Home Office. The Pharmacy Manufacturing Unit at Guy's and St. Thomas's Hospital provided escitalopram and placebo capsules. The trial was registered with Clinicaltrials.gov (identifier NCT03429075).

### Study design

**Fig. 1:**
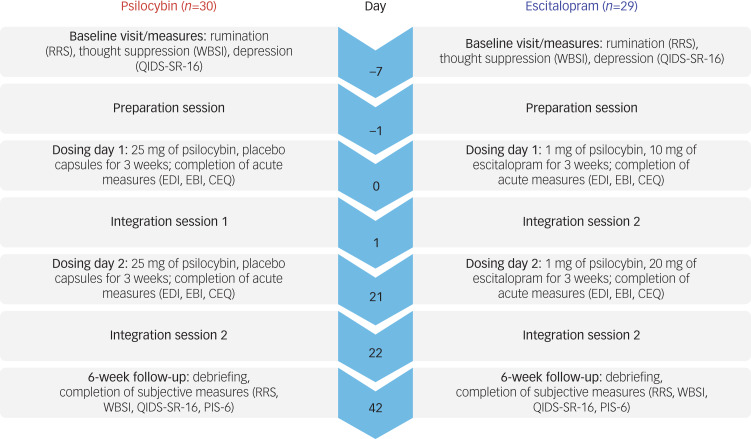
Overview of the trial procedure. Numbers indicate days from baseline (day 0) to the 6-week trial primary end-point (day 42). The listed measures are the ones included in the present study. CEQ, Challenging Experience Questionnaire; EBI, Emotional Breakthrough Inventory; EDI, Ego-Dissolution Inventory; PIS-6, Psychological Insight Scale; QIDS-SR-16, Quick Inventory of Depressive Symptomatology Self-Report; RRS, Ruminative Response Scale; WBSI, White Bear Suppression Inventory.

The full study procedure is reported elsewhere.^8^ After being randomised into two groups, all participants provided written informed consent and attended six visits over a period of 6 weeks. Visit 1 (baseline) consisted of a preparatory therapeutic session. On visit 2 (first dosing day) and visit 4 (second dosing day 3 weeks after visit 2), participants assigned to the psilocybin group received 25 mg of psilocybin and those in the escitalopram group received 1 mg of psilocybin (presumed negligible activity) to standardise expectations about receiving psilocybin and procedures attached to psychedelic therapy. Between dosing day 1 and 2, each participant received capsules and was instructed to take one each morning. Capsule ingestion increased to two each morning from the 3-week time point. The capsules contained inert filler (i.e. the inert ‘placebo’) for the participants who received 25 mg of psilocybin during visit 2, and 10 mg of escitalopram for those who received 1 mg of psilocybin during visit 2. Psychological support was provided by mental health professionals before, during and after the dosing, as well as on the integration sessions on visits 3 and 5 and optionally after the 6-week end-point at visit 6, although outcomes beyond week 6 will be presented in a forthcoming paper. An overview of the trial design is present in Figure 1.

### Participants

We used an intention-to-treat analysis. Thirty patients were randomised to the psilocybin group and 29 to the escitalopram group; constituting the entire sample from Carhart-Harris et al.^8^ Of the 59 patients enrolled, 23 (39%) were on psychiatric medication, which they stopped before starting the trial; four (7%) had to discontinue psychotherapy (see Carhart-Harris et al^8^ for stopping criteria). In the escitalopram group, four participants stopped taking their escitalopram capsules before the end of the trial because of adverse effects attributed to the drug. In the psilocybin group, one participant was smoking cannabis regularly during the trial and two participants missed the second psilocybin dosing day because of COVID-19 lockdown restrictions. The mean age was 41 years, 20 (34%) participants were women and 52 (85%) participants were White. Written informed consent was obtained from all patients. For more information on participant recruitment and demographics, see Table 1 in Supplementary Material.

### Questionnaires

#### Rumination

Rumination was measured with the RRS^14^ at baseline and at 6-week follow-up (Cronbach's alpha at baseline: 0.81; Cronbach's alpha at 6-week follow-up: 0.94). The RRS is a 22-item-self-report scale assessing ruminative tendencies by asking responders to rate how often they generally engage in ruminative thinking on a four-point scale (with 1 indicating almost never and 4 indicating almost always). The total score is obtained by summing up the 22 items, with a minimum score of 22 and a maximum of 88. Some example items from RRS include [How often do you … ] ‘think about a recent situation, wishing it had gone better’ or ‘think about how alone you feel’.

#### Thought suppression

Thought suppression was measured with the WBSI^4^ at baseline and at 6-week follow-up (Cronbach's alpha at baseline: 0.71; Cronbach's alpha at 6-week follow-up: 0.92). The WBSI is a 15 item-self-report scale assessing the tendency to generally suppress unwanted disturbing thoughts. WBSI is based on a five-point scale (with 1 indicating strongly disagree and 5 indicating strongly agree). The total score is obtained by summing up the 14 items, with a minimum score of 15 and a maximum of 75. Some example items from WBSI include ‘I always try to put problems out of mind’ and ‘I have thoughts that I try to avoid’.

#### Depressive symptoms and treatment response

Depressive symptoms were assessed with the 16-item Quick Inventory of Depressive Symptomatology Self-Report (QIDS-SR-16).^15^ The total score establishes the severity of depression, ranging from ‘absent’ (0–5) to ‘mild’ (6–10), ‘moderate’ (11–15), ‘severe’ (16–20) and ‘very severe’ (21–27). Treatment response at 6 weeks was defined as at least a 50% drop from baseline score on the QIDS-SR-16 (coded as 1 for response or 0 for no response).

#### Subjective measures relating to the psychedelic experience and successive integration

##### Acute measures

Several validated questionnaires were employed at the end of the psilocybin sessions, to retrospectively assess the acute subjective effect of psilocybin. These included the Challenging Experience Questionnaire (CEQ),^16^ Emotional Breakthrough Inventory (EBI)^17^ and the ego dissolution component from the Ego-Dissolution Inventory (EDI).^18^ With the assumption that intense acute experiences may have a larger impact on subsequent psychological change,^14^ the highest score from either of the two psilocybin dosing sessions was used for analyses. Overall, more intense acute experiences seemed to happen more frequently during dose 2 than during dose 1: 23 maximum EBI scores were reported during dose 1 and 35 were reported during dose 2; 25 maximum EDI scores were reported during dose 1 and 35 were reported during dose 2; 25 maximum CEQ scores were reported during dose 1 and 34 were reported during dose 2.

#### Psychological insights

Personal psychological insights gained after the acute psychedelic experience and successive integration were measured using the Psychological Insight Scale (PIS-6) administered at the 6-week end-point.^19^

### Statistical analyses

The data from all of the relevant time points were scored with Microsoft Excel for macOS (Microsoft Office 16) and exported for statistical analysis in RStudio (Prairie Trillium release for macOS, 2022, RStudio, Boston, USA, https://www.rstudio.com/products/rstudio/download/). All of the patients who had undergone randomisation were included in an intention-to-treat analysis. To assess the primary hypothesis, two-way mixed analyses of covariance (ANCOVAs) were performed, including RRS and WBSI scores as dependent variables, time as a within-participant effect and treatment arm (condition*)* as a between-participant effect. Baseline RRS and baseline WBSI centred scores were used as covariates to adjust for baseline differences. To assess the secondary hypothesis, three-way mixed ANCOVAs were performed, including RRS and WBSI as dependent variables, time as a within-participant effect, and condition and QIDS-SR-16 treatment response as between-participant effects. Baseline RRS and baseline WBSI centred scores were used as covariates to adjust for baseline differences. In case of significant interactions, follow-up analyses were performed with pairwise comparisons. For pairwise comparisons, effect sizes are presented as Cohen's *d*, considered to be small, medium and large above 0.2, 0.5 and 0.8, respectively.^20^ Follow-up analyses were not corrected for multiple comparisons and caution is advised when drawing inferences on them. Supportive analyses, using mixed models and non-parametric tests for follow-up comparisons, are reported in the Supplementary Material. Bivariate Pearson's correlations (two-tailed) were performed between changes in QIDS-SR-16, RRS and WBSI scores at 6 weeks compared with baseline (with Δ indicating difference in scores at 6 weeks relative to baseline). Because of normality violations for the acute measures, bivariate Spearman's rank correlations (two-tailed) were performed between the EBI, EDI, CEQ and PIS-6 and ΔRRS and ΔWBSI scores at 6 weeks relative to baseline. *P*-values <0.05 were considered statistically significant.

## Results

### Primary analysis: rumination and thought suppression

**Fig. 2:**
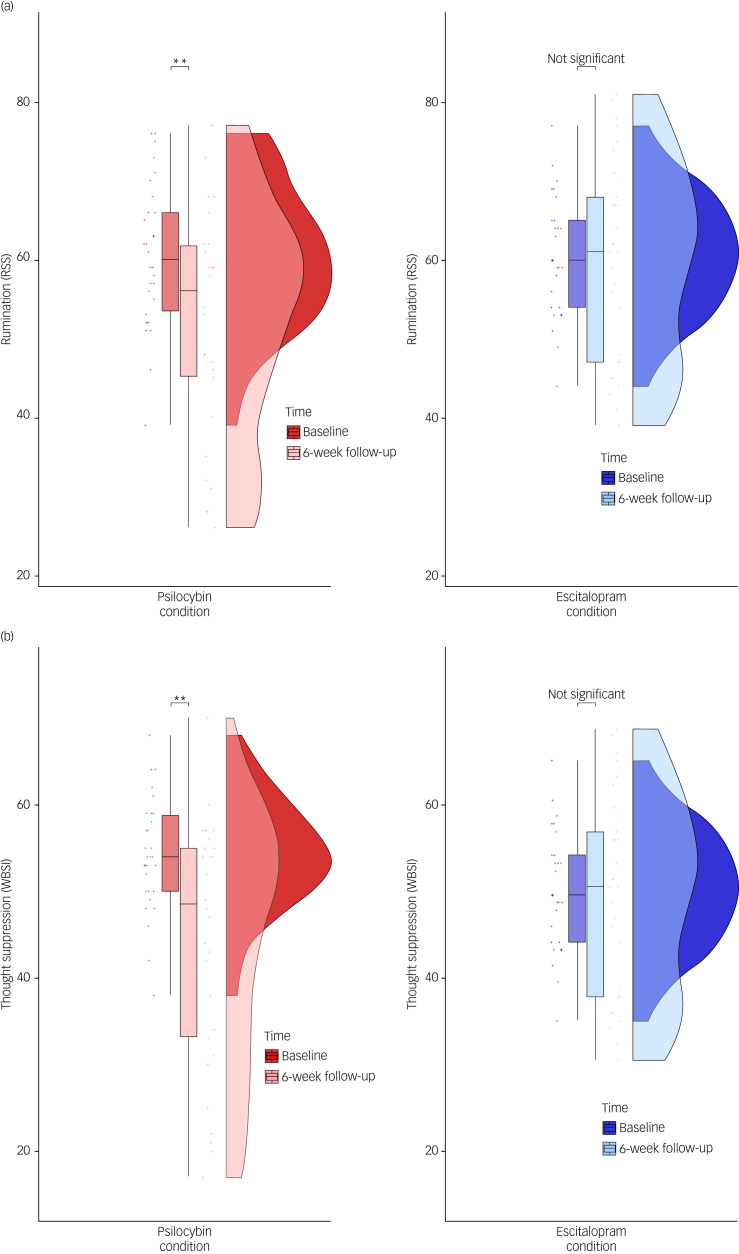
(a) Comparative effect of psilocybin and escitalopram on rumination (RRS). The plots, divided between the escitalopram and psilocybin conditions, consist of probability density plots (on the right), boxplots (on the left) and raw data points. (b) Comparative effect of psilocybin and escitalopram on thought suppression (WBSI). The plots, divided between the escitalopram and psilocybin conditions, consist of probability density plots (on the right), boxplots (on the left) and raw data points. ‘Not significant’ indicates that the difference between baseline and 6-week follow-up (time) scores is non-significant (*P* > 0.05). **The difference between baseline and 6-week follow-up (time) scores is significant, with a *P* < 0.01. RRS, Ruminative Response Scale; WBSI, White Bear Suppression Inventory.

A two-way mixed ANCOVA showed a significant main effect of time on RRS scores (F(1, 56) = 7.72, *P* = 0.007). Moreover, the analysis showed a significant time×condition interaction (F(1, 56) = 4.58, *P* = 0.037; Fig. 2(a)).

Pairwise comparisons revealed no significant differences between RRS baseline and 6-week scores in the escitalopram group (mean difference post–pre: −1.00, *P* = 0.16, *d* = 0.1), whereas in the psilocybin group, differences between RRS baseline and 6-week scores were significant (mean difference post–pre: −7.76, *P* < 0.001, *d* = 0.63). A two-way mixed ANCOVA showed a significant main within-participant effect of time on WBSI scores (F(1,57) = 19.79, *P* < 0.001), and a significant time×condition interaction (F(1,57) = 5.88, *P* = 0.019; Fig. 2(b)). In the escitalopram group, no significant differences between WBSI baseline and 6-week scores were found (mean difference post–pre: −2.85, *P* = 0.162, *d* = 0.32). In the psilocybin group, the differences between WBSI baseline and 6-week scores were significant (mean difference post–pre: −9.70, *P* < 0.001, *d* = 0.87). Mean and standard error of RRS/WBSI in the 2 groups can be found in Table 1.

**Table 1:** Descriptive statistics for the primary and secondary analysis

Descriptive statistics	Psilocybin (*n* = 30)	Escitalopram (*n* = 29)
Primary analysis		
Rumination (RRS)		
Mean (s.e.) at baseline	59.7 (2.1)	60.7 (1.5)
Mean (s.e.) at 6-week follow-up	52.7 (2.4)^a^	60.4 (1.6)
Thought suppression (WBSI)		
Mean (s.e.) at baseline	51.7 (2.1)	54.6 (1.16)
Mean (s.e.) at 6-week follow-up	44.5 (2.1)^a^	54.1 (1.1)
Secondary analysis		
Rumination (RRS) in responders	(*n* = 21)	(*n* = 14)
Mean (s.e.) at baseline	61.8 (1.83)	61.7 (2.24)
Mean (s.e.) at 6-week follow-up	49.0 (2.77)^a^	54.7 (3.39)^b^
Rumination (RRS) in non-responders	(*n* = 9)	(*n* = 15)
Mean (s.e.) at baseline	57.3 (2.79)	59.7 (2.16)
Mean (s.e.) at 6-week follow-up	61.3 (4.23)	64.2 (3.28)
Thought suppression (WBSI) in responders	(*n* = 21)	(*n* = 14)
Mean (s.e.) at baseline	54.45 (1.35)	52.7 (1.66)
Mean (s.e.) at 6-week follow-up	40.5 (2.37)^a^	51.21 (2.91)
Thought suppression (WBSI) in non-responders	(*n* = 9)	(*n* = 15)
Mean (s.e.) at baseline	53.4 (2.07)	56.4 (1.59)
Mean (s.e.) at 6-week follow-up	53.8 (3.62)	52.2 (2.81)

The outcome measures rumination (RRS) and thought suppression (WBSI) are specified for both time points, baseline and follow-up, and separately for responders (≥50% reduction of depressive symptoms) and non-responders (<50% reduction of depressive symptoms). RRS, Ruminative Response Scale; WBSI, White Bear Suppression Inventory.

a. The difference between baseline and 6-week follow-up scores is significant with a *P* < 0.01.

b. The difference between baseline and 6-week follow-up scores is significant with a *P* < 0.05.

### Secondary analysis: rumination and thought suppression in responders and non-responders

**Fig. 3:**
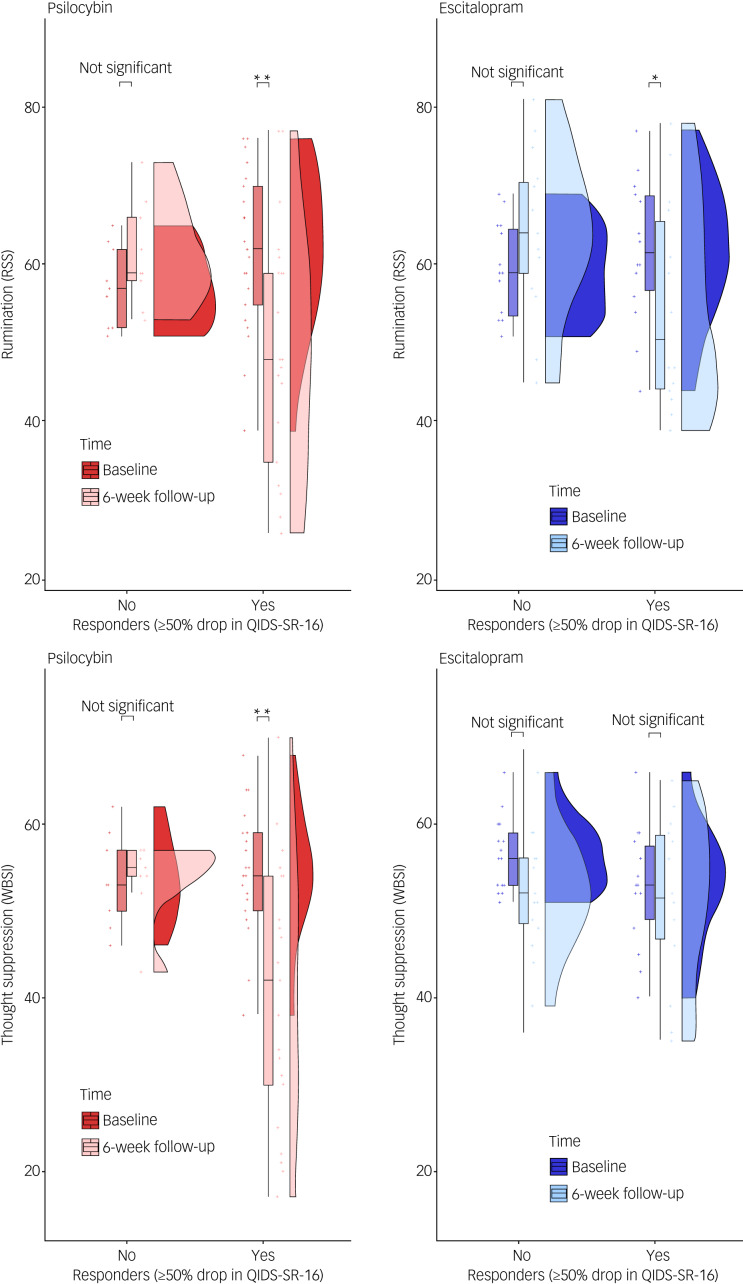
(a) Comparative effect of psilocybin and escitalopram on rumination (RRS) for both responders (≥50% drop in QIDS-SR-16 score) and non-responders (<50% drop in QIDS-SR-16 score). The plots, divided between the escitalopram and psilocybin conditions, consist of probability density plots, boxplots and raw data points. A significant time×response interaction indicated significant decreases in rumination for both psilocybin responders and escitalopram responders, whereas non-responders did not decrease in rumination in either condition. (b) Comparative effect of psilocybin and escitalopram on thought suppression (WBSI) for both responders (≥50% drop in QIDS-SR-16 score) and non-responders (<50% drop in QIDS-SR-16 score). A significant three-way interaction indicated greater decreases in thought suppression for psilocybin responders, compared with escitalopram responders, whereas non-responders did not decrease in suppression in either condition. ‘Not significant’ indicates that the difference between baseline and 6-week follow-up (time) scores is non-significant (*P* > 0.05). *The difference between baseline and 6-week follow-up (time) scores is significant, with a *P* < 0.05. **The difference between baseline and 6-week follow-up (time) scores is significant, with a *P* < 0.01. QIDS-SR-16, Quick Inventory of Depressive Symptomatology Self-Report; RRS, Ruminative Response Scale; WBSI, White Bear Suppression Inventory.

A three-way mixed ANCOVA on RRS scores revealed a significant time×response interaction (F(1,54) = 23.50, *P* < 0.001), a non-significant time×condition interaction (F(1,54) = 1.34, *P* = 0.25) and a non-significant time×condition×response interaction (F(1,54) = 0.79, *P* = 0.37; Fig. 3(a)). A significant decrease between RRS scores at baseline and 6 weeks was found for both escitalopram responders (mean difference post–pre: −7.00, *P* = 0.013, *d* = 0.62) and psilocybin responders (mean difference post–pre: −12.72, *P* < 0.001, *d* = 0.82). No significant differences for either escitalopram or psilocybin non-responders were found (*P* = 0.09 and *P* = 0.245, respectively). A three-way mixed ANCOVA on the total WBSI scores revealed a significant time×condition×response interaction (F(1,54) = 8.42, *P* = 0.005; Fig. 3(b)). Time×response and time×condition interactions were not significant (*P* > 0.05). Significant differences between WBSI scores at baseline and 6 weeks were found for psilocybin responders (mean difference post–pre: −13.95, *P* < 0.001, *d* = 0.91), but not for escitalopram responders (mean difference post–pre: −1.50, *P* = 0.575, *d* = 0.18). No significant differences for either escitalopram or psilocybin non-responders were found (*P* = 0.102 and *P* = 0.894, respectively). Mean and standard error of RRS/WBSI in the 4 groups can be found in Table 1.

### Relationship between rumination, thought suppression and depressive symptoms

In the escitalopram group, Pearson's correlations revealed a significant relationship between baseline RRS scores and WBSI scores (*r*(27) = 0.40, *P* = 0.03), and between baseline QIDS-SR-16 and both baseline RRS (*r*(27) = 0.53, *P* < 0.001) and baseline WBSI (*r*(27) = 0.37, *P* = 0.04) scores. In the psilocybin group, a significant relationship was found between baseline RRS and WBSI scores (*r*(28) = 0.48, *P* = 0.006), and between baseline QIDS-SR-16 and both baseline RRS (*r*(28) = 0.42, *P* = 0.02) and baseline WBSI (*r*(28) = 0.39, *P* = 0.04) scores. Looking at changes in the two treatment conditions, ΔQIDS-SR-16 scores in the psilocybin condition significantly correlated with both ΔRRS (*r*(28) = 0.48, *P* = 0.007) and ΔWBSI (*r*(28) = 0.49, *P* = 0.01) scores. ΔQIDS-SR-16 scores in the escitalopram condition significantly correlated with ΔRRS scores (*r*(27) = 0.39, *P* = 0.014), but not with ΔWBSI scores (*r*(27) = −0.04, *P* = 0.926). ΔRRS scores were significantly linked to ΔWBSI scores in the psilocybin condition (*r*(28) = 0.66, *P* < 0.001), but not in the escitalopram condition (*r*(27) = 0.18, *P* = 0.354).

### Impact of the subjective psychedelic experience on rumination and thought suppression

#### Acute measures during experience

**Fig. 4:**
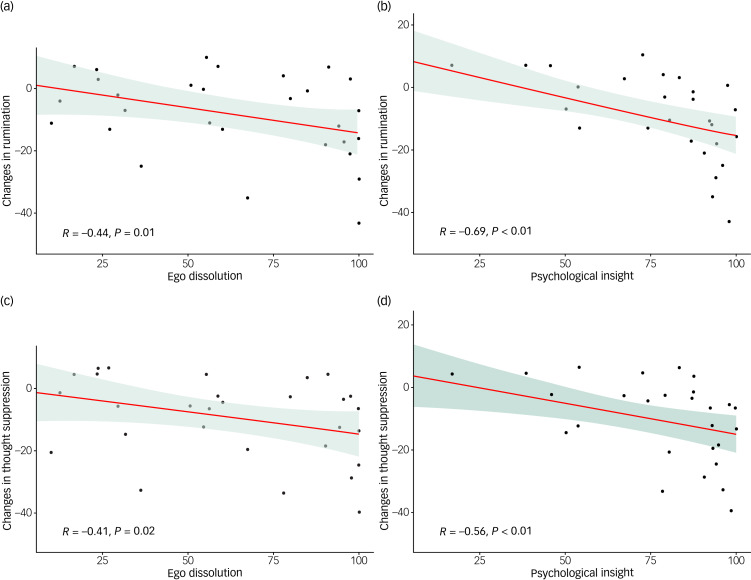
(a) Spearman's correlation (R) between the maximum ego dissolution score from patients’ two sessions (EDI) and changes in rumination (ΔRRS) in the psilocybin group. (b) Spearman's correlation (R) between psychological insights reported after the two sessions (PIS-6) and changes in rumination (ΔRRS) in the psilocybin group. (c) Spearman's correlation (R) between the maximum ego dissolution score in the two sessions (EDI) and changes in thought suppression (ΔWBSI) in the psilocybin group. (d) Spearman's correlation (R) between psychological insights after the two sessions (PIS-6) and changes in thought suppression (ΔWBSI) in the psilocybin group. EDI, Ego-Dissolution Inventory; PIS-6, Psychological Insight Scale; ΔRRS, difference in Ruminative Response Scale score at 6 weeks relative to baseline; ΔWBSI, difference in White Bear Suppression Inventory score at 6 weeks relative to baseline.

Mean scores of the acute measure in the two conditions is shown in Supplementary Table 2. In the psilocybin condition, ΔRRS scores significantly correlated with the maximum EDI score (*r*(28) = −0.44, *P* = 0.014; Fig. 4(a)). Correlations between ΔRRS score and ratings of emotional breakthrough (EBI) and challenging experience (CEQ) were not significant (*r*(28) = −0.18, *P* = 0.352 and *r*(28) = −0.01, *P* = 0.954, respectively). In the escitalopram group, no significant relationships were seen between the acute experience and changes in rumination. In the psilocybin group, ΔWBSI scores significantly correlated with the maximum EDI (*r*(28) = −0.41, *P* = 0.024; Fig. 4(b)). Correlations between ΔWBSI scores and rates of EBI and CEQ were not significant despite showing trend toward significance (*r*(28) = −0.321, *P* = 0.08, and *r*(28) = −0.349, *P* = 0.059, respectively). In the escitalopram group, no significant relationships were observed between the acute experience and changes in thought suppression.

### Insights gained during experience and successive integration

In the psilocybin group, ΔRRS scores significantly correlated with psychological insight measured with the PIS-6 (*r*(28) = −0.69, *P* < 0.001; Fig. 4(c)). Also, ΔWBSI scores significantly correlated with PIS-6 score (*r*(28) = −0.56, *P* < 0.001; Fig. 4(d)). In the escitalopram group, no significant relations between PIS-6 score and changes in rumination/suppression were present.

## Discussion

Here, we found that psilocybin had a significantly greater impact on both thought suppression and rumination than escitalopram, decreasing their severity at the 6-week primary end-point. When splitting participants into responders and non-responders, a more nuanced condition by symptom-domain pattern emerged; namely, psilocybin responders showed significant reductions in both domains, whereas the escitalopram responders only showed reductions in rumination, i.e. despite fulfilling criteria for clinical response, the domain of thought suppression remained unchanged.

### Rumination

Significant reductions in rumination at 6-week follow-up were experienced by patients classified as responders in both groups, whereas non-responders did not show decreases (Fig. 2). Reductions in rumination significantly correlated with reductions in depressive symptoms in both groups, and more participants in the psilocybin group (21 out of 30) compared with the escitalopram group (14 out of 29) were classified as responders. The present results are in line with qualitative clinical reports indicating a decrease in ruminative tendencies in patients with depression following treatment with psilocybin,^13^ as well as quantitative evidence of such improvements after successful SSRI treatment for depression.^21^

That rumination at week 6 improved in both conditions, in line with response, could imply that it is a central feature of depression that is sensitive to response to treatment, irrespective of the action of that treatment. Nevertheless, different mechanisms could be speculated to be at play in reducing rumination in the two experimental conditions. The reduction after psilocybin treatment might relate to a renewed cognitive openness and flexibility,^10^ and a decrease in avoidance-related (positive feedback) thought loops.^4,6^ Conversely, the decrease in rumination in the escitalopram responders might relate to a dampening of emotional responsivity,^11^ helping to decrease recursive negative thought loops.

### Thought suppression

Contrary to the consistent effect of both treatments on rumination, significant reductions in thought suppression at week 6 were not evident in responders to escitalopram (Fig. 3). Furthermore, unlike in the psilocybin group, changes in thought suppression did not correlate with changes in depressive symptoms in the escitalopram group, and there was no relationship between acute subjective effects and changes in thought suppression.

These results imply that, unlike for psychedelic therapy, decreased thought suppression may not be a key feature of response to SSRIs, which may decrease depressive symptoms by increasing resilience and stress tolerance.^11^ Psychedelic therapy for depression has been associated with an improved acceptance of negative memories, emotions and thoughts.^9,13^ This effect may be related to how psychedelics act on the brain to relax entrenched maladaptive patterns, enabling insights into unhealthy biases in thought and behaviour that may subsequently be relinquished.^22^ This mechanism is guided in psychedelic therapy by psychological support that surrounds the drug experience. This support helps foster key processes of insight and reconciliation. Classic serotonergic psychedelics such as psilocybin have direct agonist effects at the serotonin 2A receptor (5-HT2AR); 5-HT2AR agonism appears to dysregulate population level spontaneous neuronal activity,^23^ and 5-HT2ARs are densely expressed in high-level cortical regions. Dysregulating activity in these regions and their associated networks and circuitry may map to a dysregulation – or relaxation – of reinforced habits of mind and behaviour, and the opening of a window of plasticity for healthy psychological change.

A possible explanation for escitalopram's lack of effect on thought suppression may be related to its different pharmacology relative to psilocybin, increasing synaptic serotonin concentrations in a generalised fashion rather than targeting 5-HT2ARs directly. The increased levels of synaptic serotonin induced by SSRIs are hypothesised to have a predominant effect on stress and emotion circuitry (e.g. in limbic brain regions), dampening their responsivity through an inhibitory post-synaptic action at serotonin 1A (5-HT1A) receptors.^11^ This effect may provide resilience to stress and anxiety in depression, but may be insufficient for tackling defensive cognitive processes or avoidant defence mechanisms such as thought suppression.

### Acute subjective effects of psilocybin

In the psilocybin group, decreases in both rumination and thought suppression were shown to be related to facets of the acute psychedelic experience. Specifically, higher rates of ego dissolution, defined as the subjective experience of a compromised and dissolving sense of self,^18^ were linked with greater reductions in both rumination and thought suppression. The intensity of ego dissolution during a psychedelic experience has been previously linked to the capacity to surrender to the flow of the experience,^24^ a trait antithetical to active suppression. It is plausible to conceive of this relationship as an example of a profound altered state ‘carrying over’ into a more enduring altered trait.^25–27^

Psychological insights rated after the two psilocybin dosing sessions and successive integration visits were also positively related to reductions in both thought suppression and rumination. Such insights may be conceived of as events of clear-sightedness, facilitated by the drug-induced relaxation of biased perspectives and defensive habits.^10^ The non-significant relationship between changes in thought suppression and rumination and emotional breakthrough is surprising, as previous research has highlighted a role for intense emotional release in fostering positive therapeutic outcomes from psychedelic therapy,^17^ and it is natural to assume a relationship between acute emotional release and subsequent positive psychological change.

### Limitations

The findings of the present study should be considered in the context of some study limitations. Statistical analyses of these tertiary outcomes were neither preregistered nor adjusted for multiplicity relative to previous publications from the same RCT. Therefore, because of the potential for type 1 error, findings should be interpreted as exploratory and require replication before conclusions can be drawn.

The study population was limited in size and diversity; participants were primarily White, employed and educated, limiting generalisability. It is possible that different cultures may have divergent propensities for rumination and thought suppression, and could respond differently to the interventions examined here. It is thus important that future research include racially, ethnically and culturally diverse samples. Moreover, the secondary subgroup analysis is limited by small sample sizes. Future research should replicate the same analyses with larger sample sizes.

Both treatment groups received extensive psychological support inspired, in style, by the acceptance and commitment therapy model.^9^ Since this model focuses on increasing acceptance and reducing suppression of challenging emotions, the direct pharmacological effect of psilocybin on thought suppression and rumination cannot be separated from how it combines with therapeutic support; indeed, it is strongly hypothesised that psilocybin and context act synergistically.^28^

Despite providing the escitalopram group with a small dose of psilocybin (1 mg) to balance prior expectations, correct guessing of the psilocybin conditions seems likely, particularly in the 25 mg psilocybin arm, as acute subjective effects are typically conspicuous. Combined with differential condition-specific expectations, such correct guessing of the condition could have biased self-reported outcomes. Relatedly, for some participants, disappointment at not receiving a high dose of psilocybin may have compounded or even triggered ruminative thoughts. It is thus important that future studies carefully investigate blinding integrity and expectancy effects in the context of psychedelic-assisted therapy.

Like other SSRIs, escitalopram has been shown to have a delayed therapeutic action in treating MDD.^29^ It could be fairly argued that the 6 weeks plus 1 day course of daily escitalopram was of an insufficient duration to exploit its full potential. Supporting this view, previous work that has shown decreased rumination with SSRIs have had total trial durations of 12–14 weeks.^21^ Therefore, it cannot be ruled out that a course of escitalopram lasting longer than 6 weeks might have achieved better outcomes for rumination.

It is also worth noting that all patient-rated scales included here (i.e. the RRS, WBSI, QIDS-SR-16 and PIS-6) were administered simultaneously (at baseline and 6-week follow-up), which precludes any conclusions about the temporal effects or direction of causality between them. Thus, it remains unclear precisely how changes in rumination and thought suppression relate to improvements in depressive symptoms. Future research might consider utilising neuroimaging and additional time points to explore the possibility that changes in either or both domains (i.e. thought suppression and rumination) are key mechanisms involved in the action of either or both therapies (i.e. psilocybin and escitalopram). Moreover, one implication of poor blind integrity is that other methods, such as neuroimaging, will be required to demonstrate core treatment effects.

Lastly, despite psilocybin appearing to be safe and well tolerated in this study's population, it is important to note that the drug has mostly been investigated in small-scale clinical trials that are unable to identify uncommon but serious adverse effects. More evidence in testing the safety of psilocybin in real-world clinical populations is needed.

### Future research

Future research might consider assessing trait cognitive capacity and/or emotional regulation as a potential moderator of treatment response.^30^ Furthermore, high levels of rumination and thought suppression have been linked to obsessive–compulsive disorder, substance use disorders, eating disorders and post-traumatic stress disorder. Thus, the present results might help to explain the preliminary signs of efficacy of psilocybin in treating obsessive–compulsive disorder^31^ and substance use disorders.^32^ Future research might also offer more direct tests of certain models of the therapeutic action of psychedelics, such as relaxed beliefs under psychedelics (REBUS),^10^ and how the mechanisms they propose help to explain changes in symptoms domains such as thought suppression and rumination.

In conclusion, we discovered improvements in both rumination and thought suppression after psilocybin therapy for MDD; a more comprehensive action than was apparent for escitalopram, a first-line treatment for MDD, that had no discernible impact on thought suppression. Both rumination and thought suppression have been associated with maintenance and relapse of MDD; thus, by implication, early improvements in both rumination and thought suppression could be predictive of enduring improvements in general depressive symptoms, as previously shown.^33^

We speculate that the direct 5-HT2AR agonist action of psilocybin engages neuroplastic mechanisms that can be harnessed for therapeutic ends, potentially remediating reinforced habits of mind or behaviour that underly core pathology. More research is needed to critically appraise all aspects of psychedelic therapy, i.e. its safety, efficacy and mechanisms.

## Data Availability

The data that support the findings of this study are available from the corresponding author, T.B., upon reasonable request.

## Supplementary material

For supplementary material accompanying this paper visit http://doi.org/10.1192/bjo.2022.565. click here to view supplementary material
