# Supplementary material for: Effects of psilocybin versus escitalopram on rumination and thought suppression in depression
Source: BJPsych Open. 2022 Sep 6;8(5):e163. doi: 10.1192/bjo.2022.565 (PMC9534928; doi:10.1192/bjo.2022.565)
Supplement: Supplementary file 1 [file bjosup.zip › S2056472422005658sup002.docx]

**Supplementary Materials**

1. *Participant Recruitment*

Patients aged 18 to 80 years were recruited through trial networks, social media, and through other sources, which directed patients to a recruitment website ([www.mqmentalhealth.org/home/](http://www.mqmentalhealth.org/home/)). To be eligible, participants had to have a diagnosis of moderate to severe depression scores as indicated by a minimum score of 17 in the Hamilton Depression Rating Scale (**1**), ranging from 0 to 52. Participants were excluded if they or any immediate family members had any diagnosed psychotic disorder or other conditions unsuitable for the study. Participants with drug or alcohol dependence were also excluded. Female participants were excluded if pregnant, lactating, or planning a pregnancy. The patients discontinued any use of a psychiatric medication before trial start. Additional details about the trial exclusion criteria and participant demographics are to be found in the full study procedure reported in (**2**).

| **Table 1. Demographic and Clinical Characteristics of the Patients at Baseline*** | | |
| --- | --- | --- |
| **Characteristic** | **Psilocybin (N = 30)** | **Escitalopram (N = 29)** |
| **Demographic** | | |
| Age (range) — yr | 43.3±11.7 (21–64) | 39.1±9.7 (22–60) |
| Female sex — no. (%) | 11 (37) | 9 (31) |
| White race — no. (%)† | 28 (93) | 24 (83) |
| Employment status — no. (%) | | |
| Employed | 21 (70) | 21 (72) |
| Student | 2 (7) | 3 (10) |
| Unemployed | 7 (23) | 5 (17) |
| University level education — no. (%) | 22 (73) | 23 (79) |
| No previous psilocybin use — no. (%) | 22 (73) | 21 (72) |
| Weekly alcohol use (range) — g‡ | 36.8±43.1 (0–160) | 67.7±66.6 (0–240) |
| Discontinued psychiatric medication for trial — no. (%) | 11 (37) | 12 (41) |
| **Clinical** | | |
| Duration of illness (range) — yr | 22.1±10.7 (3–44) | 15.1±11.0 (2–46) |
| No. of psychiatric medications previously used (range) | 2.2±1.6 (0–6) | 1.8±1.5 (0–5) |
| Previous use of psychotherapy — no. (%) | 28 (93) | 26 (90) |
| QIDS-SR-16 score at pretreatment baseline (range)§ | 14.5±3.9 (7–23) | 16.4±4.1 (6–22) |
| HAM-D-17 score at pretreatment baseline (range)¶ | 19.2±2.3 (16–23) | 18.4±3.4 (11–26) |
|  |  |  |

*Plus–minus values are means ±SD. Pretreatment baseline was 7 to 10 days before dosing-day 1.*

*† Race was reported by the patients.*

*‡ To convert grams to U.K. units, divide by 8.*

*§ The scores on the 16-item Quick Inventory of Depressive Symptomatology–Self-Report (QIDS-SR-16) range from 0 to 27, with higher scores indicating greater depression.*

*¶ The scores on the 17-item Hamilton Depression Rating Scale (HAM-D-17) range from 0 to 50, with higher scores indicating greater depression. At screening, which was typically a few weeks before pretreatment baseline, all the patients had a score of at least 17 on the HAM-D-17. The depression scores reported in this table are from pretreatment baseline and not screening.*

1. *Acute measures additional scores*

***Table 2.*** *Maximum acute measures scores in the two experimental conditions (sign. differences were assessed using independent sample t-tests)*

| **Acute Measure** | **Condition** | **N** | **Mean** | **Std. Deviation** | **Sign. Differences (p<0.05)** |
| --- | --- | --- | --- | --- | --- |
| Maximum EBI | Escitalopram | 29 | 28.27586 | 25.78179 | Yes |
| Maximum EBI | Psilocybin | 30 | 69.30556 | 29.61606 |  |
| Maximum EDI | Escitalopram | 29 | 20.80172 | 22.21528 | Yes |
| Maximum EDI | Psilocybin | 30 | 60.90417 | 32.66184 |  |
| Maximum CEQ | Escitalopram | 29 | 0.941645 | 0.589122 | Yes |
| Maximum CEQ | Psilocybin | 30 | 2.062821 | 1.081248 |  |
| Maximum PIS | Escitalopram | 29 | 25.42529 | 26.87163 | Yes |
| Maximum PIS | Psilocybin | 30 | 70.26111 | 31.44288 |  |

1. *CONSORT flowchart from the original trial (Carhart-Harris et al., 2021)*

*
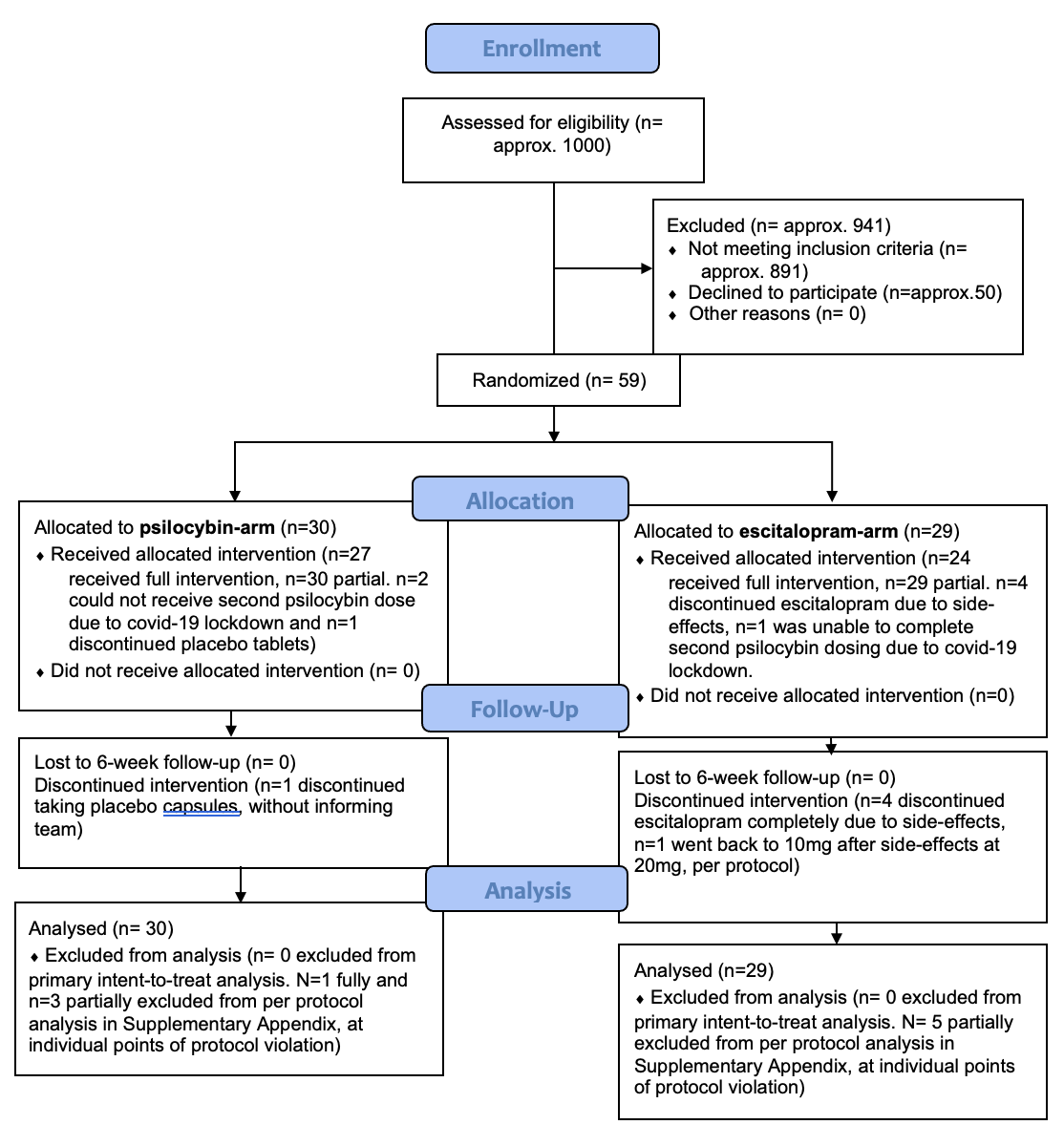
*

1. *Additional correlations (psilocybin group)*

- ΔRRS vs ego inflation (EDI-2) values (max of either session): r(28)=-0.19, p=0.31.
- ΔWBSI vs ego inflation (EDI-2) values (max of either session): r(28)=-0.2, p=0.24.
- ΔRRS vs MEQ values (max from either session): r(28)=-0.3, p=0.11.
- ΔWBSI vs MEQ values (max from either session): r(28)=-0.4, p=0.02.

1. *Rumination and thought suppression in subjects who discontinued or not SSRIs/SNRIs before entering the trial*

A total of 19 patients discontinued SSRIs or SNRIs before entering in the trial. 9 were in the escitalopram condition and 10 in the psilocybin condition. Discontinuing psychiatric medications was a prerequisite for enrolling in the trial. The washout period was of 4 weeks for fluoxetine and 2 weeks for other antidepressants. As Carhart-Harris and colleagues (**3**) previously found that patients in the psilocybin group showed greater improvement when there was no pretrial medication to discontinue, whereas the escitalopram group showed less improvement when there was no pretrial medication to discontinue, we now investigated the impact of discontinuing SSRIs on rumination and thought suppression in the two treatment arms. We also investigated the association between acute emotional breakthroughs (**5**) in the psilocybin condition and changes in thought suppression/rumination in subjects with/without SSRI taper prior to enrolment.

Results

*Main analysis*

There were no significant differences in baseline RRS or WBSI scores between the escitalopram or psilocybin condition nor between participants who discontinued SSRI/SNRIs versus those who did not (p> 0.1). A Three-Way Mixed ANOVA on RRS scores revealed a significant *time* x *condition* x *discontinuation* interaction, F(1,55) = 4.55, p = 0.037 (see Figure 2a/b). *Time* x *condition* and *time* x *discontinuation* interactions were both not significant (p> 0.08). A significant decrease between RRS-baseline/6 weeks was found for subjects in the psilocybin condition who did not discontinue SSRIs, MD Post-Pre = -9.97, p=0.02, d=0.72. No significant differences in the other conditions were found (p= 0.64, p=0.21, p=0.36). A Three-Way Mixed ANOVA on the total WBSI scores revealed a significant *time* x *condition* x *response* interaction, F(1,55)= 5.72, p= 0.03 (see Figure 2c/d). *Time* x *condition* and *time* x *discontinuation* interactions were both not significant (p> 0.1). Significant differences between WBSI-baseline/6 weeks were found for subjects in the psilocybin condition who did not discontinue SSRIs, MD Post-Pre = -12.61, p< 0.001, d=0.9. No significant differences in the other conditions were found (p= 0.63, p=0.22, p=0.07).

*
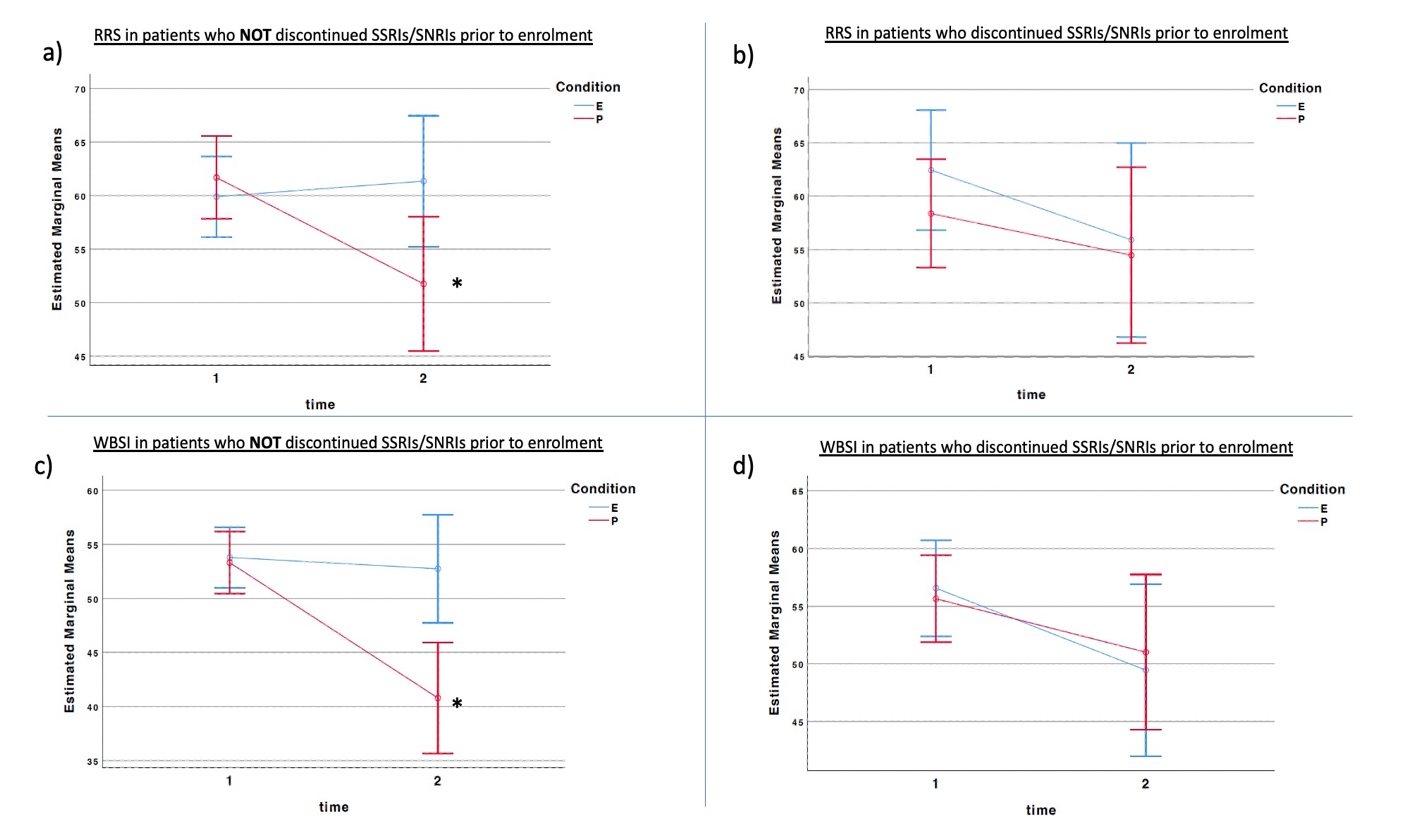
*

***FIGURE 2: (a/b) Comparative effect of psilocybin and escitalopram on RRS for participants who discontinued or not SSRIs/SNRIs before entering in the trial. (c/d) Comparative effect of psilocybin and escitalopram on WBSI for participants who discontinued or not SSRIs/SNRIs before entering in the trial.*** Time 1 indicates baseline and time 2 indicates 6-week follow-up. Dots indicate mean values, and the bars indicate 95% confidence intervals. An asterisk (*) indicates that the difference is significant with a p value <0.05.

*Association between discontinuation and emotional breakthroughs*

In the psilocybin group, participants who discontinued SSRIs reported a mean EBI of 72 (Std. Dev. = 30), participants who did not discontinue SSRIs reported a mean EBI of 67 (Std. Dev. = 29 No significant differences were found in the EBI scores between participants who discontinued and those who did not discontinue SSRIs prior to enrolment in either the psilocybin group, t(28)= 0.403, p= 0.69, or the escitalopram group, t(27)= 0.43, p= 0.66). In addition, no significant correlations were found between max EBI scores and changes in thought suppression/rumination in subjects with or without SSRI discontinuation prior to enrolment (Ps > 0.05).

Discussion

Here we showed that discontinuing SSRIs/SNRIs for at least 2 weeks before dosing session 1 differently impacted the reductions in rumination and thought suppression found in the main results of this study. Analyses were repeated after splitting the sample into subjects who discontinued all psychiatric medications versus those who were not medicated pre-trial and the same pattern of results was found (p >0.05). We found that the psilocybin group showed significant decreases in both rumination and thought suppression when there was no pretrial medication to discontinue. However, decreases were no longer significant in the sub-sample who discontinued SSRI/SNRI (or any psychiatric) medication. In the escitalopram group, discontinuing SSRI/SNRIs did not influence rumination and suppression as both subgroups did not present significant decreases. The results are somewhat in line with previously reported results from the trial (**1**), which showed that depression scores in the psilocybin group were greater in a sub-sample who were not medicated coming into the trial. There do not, however, replicate the finding of greater improvements in discontinuers (versus non-discontinuers) in the escitalopram arm.

We did not find any significant difference in the EBI scores (**5**) between participants who discontinued SSRI/SNRIs and those we did not, in either the psilocybin group or the escitalopram group. Focusing on the psilocybin group, we looked at correlations between EBI (acute) and thought suppression/rumination (post-acute change) in subjects with or without SSRI discontinuation prior to enrolment, but we did not find any notable differences in the correlations between these variables in these sub-groups. It therefore appears that tapering SSRIs before exposure to psilocybin does not affect the ability of having emotional breakthrough experiences under psilocybin. Another explanation could be linked to SSRI discontinuation syndrome (**4**). Acute withdrawal effects after discontinuing SSRIs are common and are speculated to last for several weeks. Withdrawal symptoms might mimic the symptoms for which drugs are initially prescribed - and for this reason, participants who discontinued SSRI/SNRIs might have engaged in rumination focused on these symptoms, negatively impacting the results for that subgroup.

1. *References*
2. Hamilton M. A rating scale for depression. Journal of Neurology, Neurosurgery & Psychiatry. 1960;23(1):56-62.
3. Carhart-Harris R, Giribaldi B, Watts R, Baker-Jones M, Murphy-Beiner A, Murphy R, Martell J, Blemings A, Erritzoe D, Nutt DJ. Trial of Psilocybin versus Escitalopram for Depression. *N Engl J Med*. 2021 Apr 15;384(15):1402-1411.
4. Carhart-Harris R, Giribaldi B, Watts R, Baker-Jones M, Murphy-Beiner A, Murphy R, Martell J, Blemings A, Erritzoe D, Nutt DJ. Psilocybin for Depression [Response to Letters to the Editor]. N Engl J Med. 2021 Aug 26;385(9): 862-864.
5. Cipriani A, Furukawa TA, Salanti G, Chaimani A, Atkinson LZ, Ogawa Y, Leucht S, Ruhe HG, Turner EH, Higgins JPT, Egger M, Takeshima N, Hayasaka Y, Imai H, Shinohara K, Tajika A, Ioannidis JPA, Geddes JR. Comparative efficacy and acceptability of 21 antidepressant drugs for the acute treatment of adults with major depressive disorder: a systematic review and network meta-analysis. Lancet. 2018 Apr 7;391(10128):1357-1366.
6. Roseman L, Haijen E, Idialu-Ikato K, Kaelen M, Watts R, Carhart-Harris R. Emotional breakthrough and psychedelics: Validation of the Emotional Breakthrough Inventory. Journal of Psychopharmacology. 2019;33(9):1076-1087.
7. Lerner A, Klein M. Dependence, withdrawal and rebound of CNS drugs: an update and regulatory considerations for new drugs development. Brain Commun 2019;1(3):fcz025.
